# Dynamic Structure-Based Pharmacophore Model Development: A New and Effective Addition in the Histone Deacetylase 8 (HDAC8) Inhibitor Discovery

**DOI:** 10.3390/ijms12129440

**Published:** 2011-12-19

**Authors:** Sundarapandian Thangapandian, Shalini John, Yuno Lee, Songmi Kim, Keun Woo Lee

**Affiliations:** Division of Applied Life Science (BK21 Program), Systems and Synthetic Agrobiotech Center (SSAC), Plant Molecular Biology and Biotechnology Research Center (PMBBRC), Research Institute of Natural Science (RINS), Gyeongsang National University (GNU), 501 Jinju-daero, Gazwa-dong, Jinju 660-701, Korea; E-Mails: sunder@bio.gnu.ac.kr (S.T.); shalini@bio.gnu.ac.kr (S.J.); youknow@bio.gnu.ac.kr (Y.L.); skim@bio.gnu.ac.kr (S.K.)

**Keywords:** molecular dynamics simulation, pharmacophore, virtual screening, Lipinski’s rule, molecular docking

## Abstract

Histone deacetylase 8 (HDAC8) is an enzyme involved in deacetylating the amino groups of terminal lysine residues, thereby repressing the transcription of various genes including tumor suppressor gene. The over expression of HDAC8 was observed in many cancers and thus inhibition of this enzyme has emerged as an efficient cancer therapeutic strategy. In an effort to facilitate the future discovery of HDAC8 inhibitors, we developed two pharmacophore models containing six and five pharmacophoric features, respectively, using the representative structures from two molecular dynamic (MD) simulations performed in Gromacs 4.0.5 package. Various analyses of trajectories obtained from MD simulations have displayed the changes upon inhibitor binding. Thus utilization of the dynamically-responded protein structures in pharmacophore development has the added advantage of considering the conformational flexibility of protein. The MD trajectories were clustered based on single-linkage method and representative structures were taken to be used in the pharmacophore model development. Active site complimenting structure-based pharmacophore models were developed using Discovery Studio 2.5 program and validated using a dataset of known HDAC8 inhibitors. Virtual screening of chemical database coupled with drug-like filter has identified drug-like hit compounds that match the pharmacophore models. Molecular docking of these hits reduced the false positives and identified two potential compounds to be used in future HDAC8 inhibitor design.

## 1. Introduction

**Figure 1:**
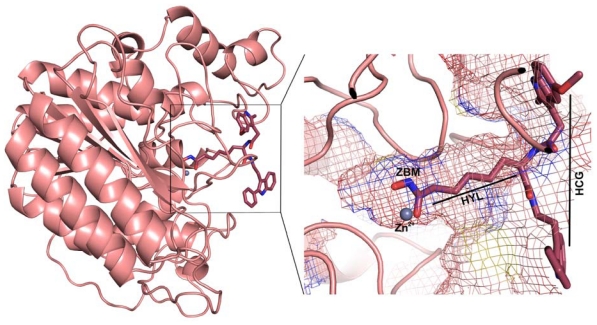
The 3D structure of human histone deacetylase 8 (HDAC8) bound with an inhibitor (PDB code 2V5X). A zoomed view of inhibitor binding region shows the tunnel-like active site in mesh form. The hydroxamic acid part of inhibitor binds close to the metal (Zn^2+^) ion whereas the aliphatic chain and hydrophobic cap groups occupy the tunnel and surface of the active site.

Histone deacetylase (HDAC) is a zinc-dependant enzyme involved in the deacetylation of terminal acetylated lysine residues of histone proteins [1–3]. Deacetylation is one of the post-translational chromatin modifications that regulate the epigenetic control of gene expression. This chromatin modification is indispensable as this prepares the chromatin accessible to a large number of chromatin interacting proteins [4]. The acetylation and deacetylation of lysine residues of histone protein are controlled by histone acetyl transferase (HAT) and HDAC enzymes, respectively [5,6]. The mechanism behind controlling the gene transcription is the extent of histone-DNA binding which is the effect of exposure of the positive charge of lysine residues on deacetylation. In contrast, HATs loosen the histone-DNA binding by acetylating the positively charged lysine residues back to their acetylated form and leads to the transcriptional activation [7]. The transcriptional repression of various pre-programmed set of genes including tumor suppressor gene leads to cancer. These repression and activation of transcription have fundamental regulatory roles in developmental processes and their deregulation has been linked to the progression of cancers and different human disorders [8]. HDAC inhibitors can induce cancer cell death whereas normal cells are relatively resistant to the inhibitor-induced cell death [9,10]. HDACs are present in almost all organisms and at least, to date, 18 types of HDACs were identified and classified into four broad classes. First 11 of these 18 types are named HDACs, which contain divalent Zn^2+^ cation as catalytic machinery and the rest of them are known as sirtuins, which are NAD-dependant enzymes due to their deacetylation mechanism. Class I HDACs include HDAC1-3 and 8 whereas Class II is made of HDACs 4–7, 9 and 10. All of the sirtuins are grouped into Class III HDACs as their mechanism of deacetylation process is different to that of other HDACs. HDAC11, which shares substantial homology with Class I and Class II HDACs, is the only member of Class IV HDACs. Because of the widespread biological effects of HDACs the inhibition of these enzymes has emerged as a new therapeutic approach to treat many diseases such as neurodegenerative, hereditary, inflammatory diseases and cancer [11–18]. HDAC inhibitors are a structurally distinct class of chemical compounds with unique structural elements to compliment the active site components. The generally known pharmacophoric elements present in HDAC inhibitors are: (i) a zinc-binding moiety (ZBM) to co-ordinate with the catalytic metal ion; (ii) a hydrophobic cap group (HCG) to bind at the surface of the tunnel-like active site; and (iii) a hydrophobic linker (HYL) that joins both ZBM and HCG. This hydrophobic linker is normally six carbon chains longer, which is almost equal to the length of the tunnel of the active site (Figure 1) [7].

To date, a number of potential HDAC inhibitors are in clinical trials. Suberoylanilide hydroxamic acid (SAHA) was the first HDAC inhibitor approved by FDA. In terms of diverse chemical structures, HDAC inhibitors include a wide range of scaffolds and can be classified into structural classes such as aliphatic acids, hydroxamic acids, cyclic peptides and benzamides. Design and evaluation of linkerless hydroxamic acid derivatives reported that these new linkerless scaffolds have shown >100 fold selectivity for HDAC8 over other Class I and II HDACs. In addition, it is also reported that the active site of HDAC8 is unconventionally malleable and can accommodate inhibitors of diverse structures with no conventional structural arrangement, which is “ZBM-linker-HCG” [19]. In terms of selectivity of inhibitors, modifications of the ZBM, linker, and HCG have individually contributed to the selective nature of the compounds. All these modifications at various positions have led to the class-selective rather than isoform-selective inhibitors [19,20]. Various experimental evidences reported that different subtypes have intrinsic differences in substrate selectivity [20–22]. A clear knowledge of the selectivity profile over various HDACs from the structural perspective is necessary to design isoform-selective HDAC inhibitors with elevated potency [20]. All Zn-dependant HDACs possess well-conserved deacetylase core domains of approximately 400 amino acids and identical catalytic machinery. This functionally important catalytic machinery includes Zn (a divalent cation) and a charge relay system formed by two histidine and two aspartate residues very close to the metal ion. The active site region of HDACs is highly conserved among the family members and making it very difficult to design isoform-selective inhibitors. A recent study reported that the exposed surface of the active site entrance is less conserved among HDAC isoforms and can be utilized in designing isoform selective inhibitors [20]. Functional HDACs, except HDAC8, are usually found as high molecular weight multiprotein complexes and their purified recombinant forms are also enzymatically inactive [23]. Therefore, HDAC8 has been considered as a best model among mammalian HDACs from a structural biology perspective. The first crystal structure of HDAC8 was determined with a bound inhibitor in 2004 followed by some other diverse inhibitors and with mutations [24–28]. A study observing the structural differences due to the bound inhibitor has reported that the active site topology showed large differences based on the nature of the co-crystallized inhibitors. It also concluded that the enzyme’s inhibition is strongly regulated by the surface malleability [29]. Though the differences in the crystal structures showcase interesting information that they can be used in designing new class of selective inhibitors, the conformational flexibility of the enzyme also has become indispensable to be considered. Thus the utilization of molecular dynamic (MD) simulations to consider the flexibility of protein in the development of pharmacophore models can be a right choice and improve its reliability [30,31].

In this study pharmacophore models were developed using the representative structures of HDAC8 obtained from MD simulations. Two inhibitors showing potent inhibitory profile for HDAC8 were selected and docked into the active site of the enzyme to prepare enzyme-inhibitor complexes. The MD simulations of these complexes were performed and the representative structures from the highly clustered conformations of MD simulation trajectory were selected to be used in the development of pharmacophore models. These pharmacophore models, after validation, were used in virtual screening to identify new and potential hits for future drug design. Molecular docking study has reduced the probability of picking false positives as potential hits. Finally we obtained two potential compounds which can be used in future HDAC8 inhibitor design.

## 2. Results and Discussion

### 2.1. Molecular Dynamic Simulation Studies

**Figure 2:**
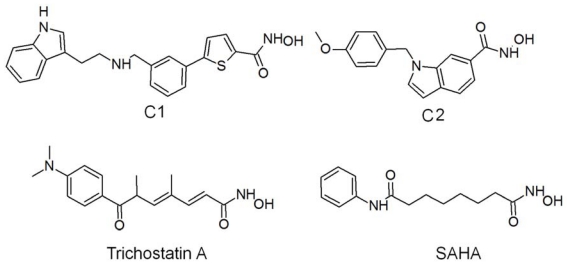
The 2D chemical structures of HDAC8 inhibitors used in this study are displayed with two clinically proven HDAC inhibitors for structural comparison.

Two most active HDAC8 inhibitors were selected from a set of inhibitory molecules with the experimental activity values determined using the same biological assay procedure collected from the literature. These two compounds are structurally very different except having the hydroxamic acid moiety as common zinc binding group and showing very similar HDAC8 inhibitory profile. The C1 has the modified linker portion when compared to trichostatin A and SAHA whereas the C2 is linkerless (Figure 2). Thus these two compounds were selected to be used in MD simulation studies and the dynamic structures of HDAC8 complexed with these two diverse inhibitors were used in structure-based pharmacophore modeling. Utilization of dynamic structures of the enzyme obtained upon binding of two diverse most active inhibitors in pharmacophore development has an advantage over using a single static conformation of the enzyme [31]. These compounds were docked into the active site of HDAC8 enzyme using GOLD (Genetic Optimization for Ligand Docking) 4.1 program [32]. The X-ray determined crystal structure (PDB code: 2V5X) was used in molecular docking study. The binding poses of the inhibitors were carefully chosen as it is very important that the metal binding part of the inhibitors should be located close to the divalent metal (Zn^2+^) ion (Figure S1). The best binding conformation among the poses predicted by GOLD program was selected and the protein-inhibitor complex is used in 5 ns MD simulations. Before selecting the crystal structure with the PDB code 2V5X for further study, another HDAC8 crystal structure (PDB code: 1T64) was considered in molecular docking as it is the high resolution structure resolved so far. The 1T64 was identified to have two adjacent active sites bound with two molecules of inhibitors but only one site contains the metal ion responsible for the catalytic activity [20]. The best docked poses of inhibitors bound at this metal containing active site were selected and subjected to MD simulation for 5 ns (data not shown).

**Figure 3:**
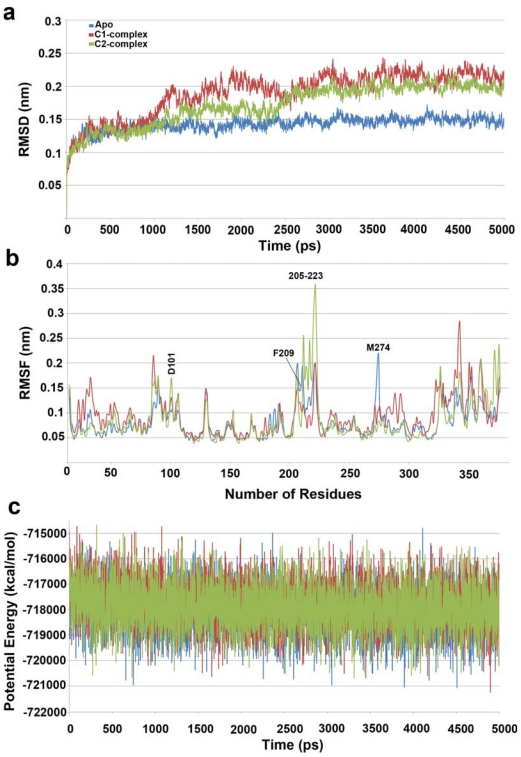
Comparison of (**a**) root mean square deviation (RMSD) of backbone atoms; (**b**) root mean square fluctuation (RMSF); and (**c**) potential energy values of 5 ns molecular dynamic (MD) simulations for three systems.

The results of MD simulation have displayed only a single active site merging both the initial active sites. This behavior of 1T64 was considered unfavorable and thus 2V5X, which exhibits a single active site in its crystal structure, was selected for this study. Another MD simulation of HDAC8 without inhibitor (apo-form) was also performed to compare the changes upon the binding of inhibitor. Our simulation results were analyzed using tools bundled with GROMACS distribution package. The root mean square deviation (RMSD) of backbone atoms of the protein revealed that apo-form of HDAC8 was very stable with an average RMSD value of 0.14 nm throughout the simulation time whereas the inhibitor complexes were slightly unstable during the first half of the simulation time *i.e.*, up to 2500 ps. During the second half of the simulation time (2500–5000 ps), the RMSD values for HDAC8-C1 and HDAC8-C2 complexes were maintained at an average value of 0.19 and 0.17 nm, respectively. The RMSD value for HDAC8-C2 complex was slightly lower than HDAC8-C1 complex. This difference can simply be explained upon the size and chemical differences between both of them. These changes in RMSD also conclude that the binding of inhibitor seems to increase the conformational flexibility of HDAC8. None of the three MD simulations resulted in very unstable structures which in turn confirmed the convergent behavior of the systems during the entire course of simulation and the results are reliable for further studies (Figure 3a).

The root mean square fluctuation (RMSF) plot was plotted to estimate the changes in dynamic flexibility of the regions of protein structure due to the inhibitor binding. It also has confirmed that all the catalytically important residues including charge relay system and most of the tunnel and surface forming residues except F209 and M274 were very stable throughout the simulation in all systems. The D101 which is present at the surface of the active site has shown increased flexibility in the C2 complex whereas the flexibility of F209 and M274 has reduced upon inhibitor binding (Figure 3b). The potential energy values of all the systems have shown a smooth decrease till 500 ps and later they stabilized around a constant value of −718,000 kcal/mol (Figure 3c).

**Figure 4:**
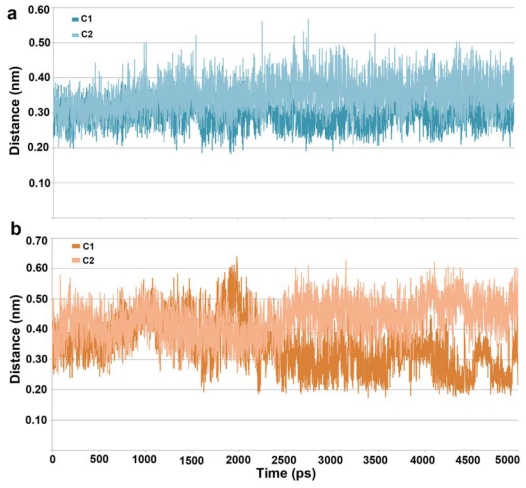
Distance between the hydroxamic acid moieties of inhibitors and two catalytically important histidine residues (**a**) H142 and (**b**) H143 in the mechanism of HDAC8 enzyme.

The distance between the hydroxamic acid moieties of bound inhibitors and the catalytic divalent (Zn^2+^) metal ion present at the bottom of the tunnel like active site was measured throughout the simulations. The average distance between the hydroxamic acid moieties of both inhibitors and Zn^2+^ was 0.19 nm which was maintained very stable during the simulation (Figure S2). Both the inhibitors were in interacting distance from the divalent metal Zn^2+^ ion and the charge relay system. A charge relay system comprising a couple of histidine (H142 and 143) and aspartate (D178 and 183) residues was identified as very important tool in deacetylation process [33–35]. Some structural studies predicted that both histidine residues forming two independent hydrogen bonds are essential for the charge relay systems present in the active site [1,36]. In order to confirm the presence of the inhibitory molecules within interacting distance from the functionally important residues H142 and H143, we have calculated the distance between the hydroxamic acid moieties of the inhibitors and these histidine residues. From the distance plot, we observed that C1 stayed closer to both the histidine residues when compared to C2. In detail, hydroxamic acid moieties of both the compounds were in similar distance with the average values of 0.30 and 0.34 nm from H142 whereas the distance between hydroxamic acid moieties of both compounds and H143 was fluctuating. The center of mass of six atoms of hydroxamic acid moiety and the corresponding histidine residues were used in these distance calculations. The average distance values observed between the hydroxamic aid moieties of compounds (C1 and C2) and H143 (Figure 4a,b) were 0.34 and 0.43 nm, respectively. As the hydrogen bond interaction is important for any HDAC8 inhibitors we calculated the hydrogen bond interactions between the inhibitors and protein during the course of simulation. The C1 and C2 have formed 2 and 1 hydrogen bonds as average values throughout the simulation time with maximum of four and two hydrogen bonds with the active site residues, respectively (Figure S3). Based on the size of the compounds, it was assumed that C1 binds the whole stretch of the tunnel-like active site using its hydroxamic acid, thiophenyl, phenyl and four-membered alkylamino chain while positioning its indole moiety outside the surface of the active site for extended interactions. In the other hand, the C2 positions itself only within the tunnel-like active site approaching the hydrophobic surface with its phenylmethoxy end (Figure S1). An analysis over the orientation of C1 throughout the simulation time has shown that its phenyl ring was placed against the phenyl ring of F139 enabling π-π interaction during the simulation time (not shown in figure). The orientation analysis on C2 has shown that six-membered part of its indole ring was stacked between F139 and H180 enabling very strong hydrophobic interactions at the active site. Based on the results of these analyses both the compounds were predicted to be interacting well at the active site and thus their middle structures snapped from the clustering analysis were used in structure-based pharmacophore modeling.

**Figure 5:**
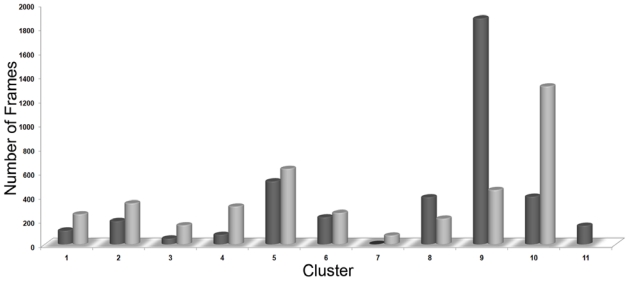
The histogram obtained from the clustering analyses of last 4 of 5 ns conformational regions of HDAC8-inhibitor complex MD simulation trajectories. Dark and light gray color cylinders represent the clusters obtained from HDAC8-C1 and HDAC8-C2 complexes, respectively.

**Figure 6:**
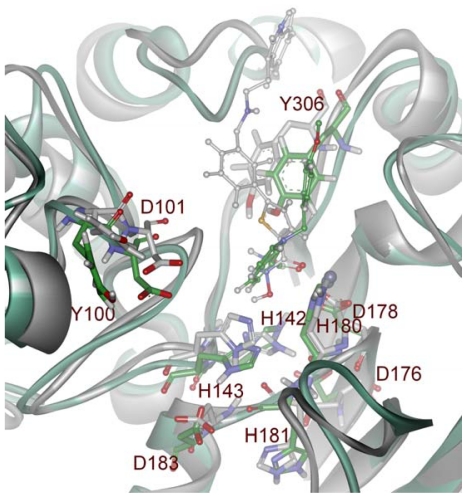
Overlay of the representative structures obtained from 5 ns MD simulations of HDAC8-C1 (white) and HDAC8-C2 (green) complexes. Important amino acid residues of HDAC enzyme and the inhibitors are shown in stick and ball-stick forms, respectively.

The results of the simulation of HDAC8 enzyme with two different inhibitors were further analyzed using clustering method to cluster the snapshots based on the RMSDs of atom positions. Each structure is added to a cluster when its distance to any element is less than a given cutoff. This analysis will be useful to statistically characterize conformational families which are sampled during the simulations. Clusters were obtained with a cut off value of 0.077 nm and each cluster has a representative structure. The simulation with C1 and C2 were divided into 11 and 10 clusters, respectively, based on the RMSD values of the atom positions (Figure 5). This clustering process also calculates a middle structure for every single cluster obtained. In terms of simulation with C1, the ninth cluster containing 47% of simulated conformations was selected and its middle structure was obtained for further study. In the other hand from the simulation result of C2, tenth cluster containing 33% of the simulation structures was selected and its middle structure was taken to be used in further study. The representative middle structures from both the simulations with bound inhibitor were obtained and superimposed to observe the conformational changes occurred among active site catalytic residues including charge relay system upon different inhibitor binding. All of the catalytically important residues except H143, which is slightly tilted from its position, have behaved same for both the inhibitors whereas the surface forming hydrophobic residues such as Y100 and Y306 have fluctuated differently as response to the nature of the bound inhibitor (Figure 6).

### 2.2. Structure-Based Pharmacophore Models

**Figure 7:**
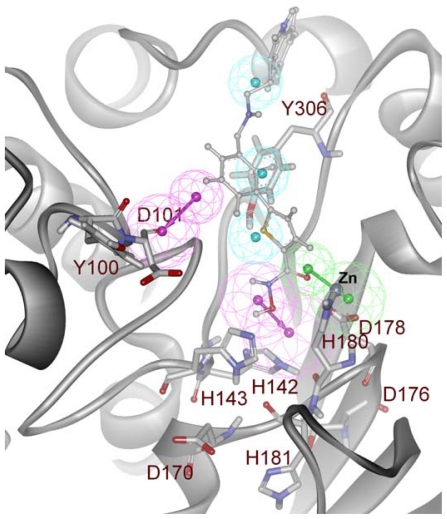
Structure-based pharmacophore model generated from the HDAC8-C1 complex. Secondary structure of protein is shown in cartoon and amino acid residues are shown in stick form. The C1 inhibitor is shown in ball-stick representation. The identified pharmacophoric features are shown in green, cyan and magenta for HA, HY and HD features, respectively.

Pharmacophore models were generated using the two representative structures obtained from the MD simulations of HDAC8 bound C1 and C2, respectively. The hydrogen bond acceptor (HA), hydrogen bond donor (HD), and hydrophobic (HY) chemical features were generated based on the interaction points available from the active site. Based on the mechanism of action and the general pharmacophore for HDAC8 inhibitors, residues were chosen and to trim down the generated pharmacophoric features we have selected only the features that are generated complementary to these residues. Seven pharmacophoric features containing three HD, three HY, and one HA were selected as a pharmacophore model (Pharm-A) from the dynamic structure resulted from HDAC8-C1 complex. Three HD features of Pharm-A were generated as complementary features to D101, H142 and D178 residues. Two features complementing H142 and D178 were located very close to each other and thus were merged to create a new average feature at their average position. The only HA feature of Pharm-A was generated as a complimentary to the catalytic metal (Zn^2+^) ion whereas the three HY features were picked to compliment the hydrophobic tunnel and surface residues. Thus the final Pharm-A contains one HA, two HD and three HY features was generated from HDAC8-C1 complex (Figure 7).

**Figure 8:**
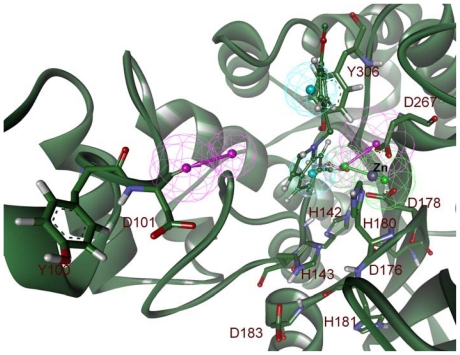
Structure-based pharmacophore model generated from the HDAC8-C2 complex. Secondary structure of protein is shown in cartoon and amino acid residues are shown in stick form. The C2 inhibitor is shown in ball-stick representation. The identified pharmacophoric features are shown in green, cyan and magenta for HA, HY and HD features, respectively.

**Figure 9:**
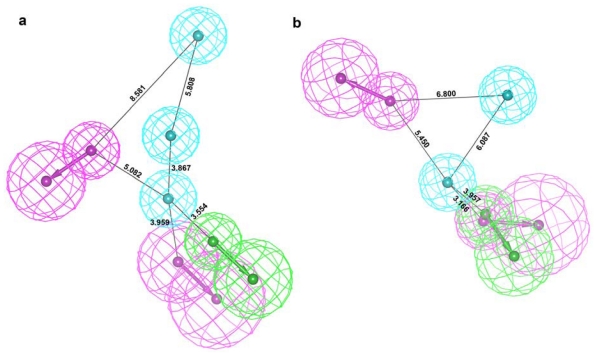
Generated pharmacophore models (**a**) Pharm-A and (**b**) Pharm-B are shown with their inter-feature distance constraints. Green, cyan, and magenta colors represent HA, HY and HD features, respectively.

In addition to the Pharm-A, another pharmacophore model was developed using the middle structure obtained from the simulation of HDAC8 bound with C2. Similar to the generation of Pharm-A, the pharmacophoric features generated complimentary to the important active site components were selected and developed a pharmacophore model (Pharm-B). Initially this pharmacophore model was made of one HA, two HY, and four HD features. The HA and HY features of Pharm-B were the complimentary features to the metal ion and the tunnel as well as surface forming residues. Four HD features were the complimentary result to D101, D178, D267, and Y306 residues of the active site. Three of these four HD features were formed very close to each other and has the starting vector point at the same place. Thus these three HD features were merged together to form an average HD feature. The final Pharm-B contains five pharmacophoric features which include one HA, two HD, and two HY features (Figure 8). These generated pharmacophore models represent the flexibility of protein and ligands in its conformational space. Comparison of the generated pharmacophore models revealed that both the models contain same pharmacophoric features except an additional HY feature in Pharm-A. Although the models look similar in their chemical features they are different in their 3D conformational space and this conformational difference is considered as the major advantage of the dynamic structure-based pharmacophore models (Figure 9).

### 2.3. Validation of Generated Pharmacophore Models

The generated pharmacophore models were validated for their reliability to be used in database screening and prediction of new compounds. Two different methods with different information were employed in validating the pharmacophore models. The first method is based on the best binding orientation used in MD simulations while the second method is based on the collected dataset of HDAC8 inhibitors with the experimental inhibitory profile. In the first validation method, the best binding orientations of C1 and C2 were matched with generated pharmacophore models A and B, respectively. This study has shown interestingly that both the compounds could map the pharmacophoric features well except one HD feature generated complimentary to D101.

In terms of C1, its terminal indole, central phenyl, and thiophene rings mapped upon the HY features of Pharm-A, whereas the closely present functional groups of hydroxamic acid have mapped over the HA and HD features (Figure S4). Comparing to this, C2 has mapped the two HY features of Pharm-B with its phenyl and indole rings whereas the hydroxamic acid moiety mapped over the HA and HD features (Figure S5). In the second method, a dataset containing 100 HDAC8 inhibitors with experimental activity values was screened using the generated pharmacophore models as 3D structural queries. This dataset was divided into three categories: (i) active (<0.1 μM); (ii) moderately active (≥0.1 <1 μM); and (iii) inactive (>1 μM) to assess the predictive ability of the pharmacophore models. Compounds of this dataset were screened using the pharmacophore models. Of 100 compounds, Pharm-A and Pharm-B have screened 52 and 57 compounds, respectively. Pharm-A has identified 15 of 17 active compounds, 35 of 68 moderately active compounds, and only 2 of 15 inactive compounds with the percentage values of 88.24, 51.47, and 13.33, respectively. Pharm-B has identified 13 of 17 active compounds, 40 of 68 moderately active compounds, and 4 of 15 inactive compounds with the percentage values of 76.47, 58.82, and 26.66, respectively (Table 1). In addition, another pharmacophore model (Pharm-X) was developed from the X-ray structure (PDB code: 2V5X) directly to be kept as control to evaluate the results of Pharm-A and Pharm-B. This Pharm-X was used in the same way as other models in screening the test set of 100 known HDAC8 inhibitors. This control pharmacophore model has screened the known set of compounds with lesser predictions compared to that of Pharm-A and Pharm-B models (Table 1). These statistical results from the validation process proved that the generated pharmacophore models are good enough to be used in database screening to find new lead compounds with great potential to inhibit HDAC8.

### 2.4. Database Screening

Virtual database screening method using pharmacophore models as 3D queries has successfully been used in retrieving potential compounds that can confidently be used in novel drug discovery and development. As this approach serves an advantage over any *de novo* design methods by providing a set of compounds directly for the biological testing and it is very popular among drug discovery scientists. Both the validated pharmacophore models were used as 3D queries in database screening. A chemical database named Asinex containing 213,462 compounds was utilized in database screening procedure. The chemical compounds of the database fitting with all the pharmacophoric features of Pharm-A and Pharm-B were identified through ligand pharmacophore mapping process along with the *Best/Flexible* search option. During database screening *Maximum Omitted Features* option was set to “0” to screen the databases for the compounds those fit on all pharmacophoric features of Pharm-A and Pharm-B. The first pharmacophore model, Pharm-A, has identified 627 compounds mapping all of its pharmacophoric features. The hit compounds resulted from this step were considered in Lipinski’s drug-like screening which resulted 515 compounds as Lipinski positives. These compounds were further filtered based on the fit value of the most active compound in the experimental dataset used in validation process. The most active compound (C1) has scored a fit value of 2.02 mapping five of six features of Pharm-A missing only the HD generated against D101. Thus 49 compounds mapping all the features and scoring a fit value greater than 2 were selected as hits from database screening using Pharm-A. Adding to these hits, the second pharmacophore model, Pharm-B, was also used in database screening to identify more hit compounds. Pharm-B containing five features has identified 2753 compounds mapping all of five features. These compounds were subjected to drug-like screening based on Lipinski’s rule which has identified 2386 compounds as Lipinski positives. Based on the fit value of the most active compound (C1) for Pharm-B, which is 3.7, the hit compounds were filtered. Filter based on the fit value has identified 51 compounds which mapped all the features of Pharm-B and scored a fit value greater than C1. Totally 100 compounds were identified, 49 from Pharm-A and 51 from Pharm-B, respectively, through database screening and subsequently considered in molecular docking study. The 33 of these 100 compounds were identified by both the pharmacophore models and thus contains the characteristics of both C1 and C2 inhibitors.

### 2.5. Molecular Docking

**Figure 10:**
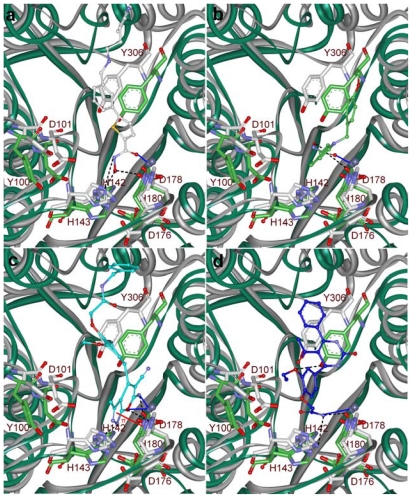
Binding modes and molecular interactions of (**a**) inhibitor C1 (**b**) inhibitor C2 (**c**) hit 1 and (**d**) hit 2 at the active sites of two different inhibitor-induced conformations. White and green cartoons represent C1- and C2-induced conformations of HDAC8 enzyme. Amino acid residues are shown in stick form whereas the ligands are shown in ball-stick form. Hydrogen atoms are not shown for clear view.

**Figure 11:**
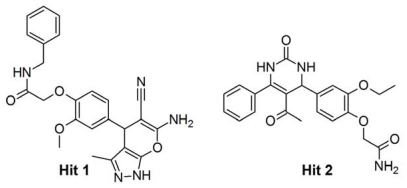
The 2D chemical structures of identified hits.

Final hit compounds along with the most active C1 and C2 were docked in to the active site of HDAC8. The prepared middle structures obtained from the MD simulations with both most active compounds C1 and C2 were used as target protein molecules. The molecular docking results were used as a post-docking filter to select the compounds those interact with the active site amino acids and to predict the binding orientations of the hit compounds. The docking program GOLD has generated several feasible binding conformations for each compound and ranked them according to their fitness scores. The bound conformation with the most favorable energies was considered as the best binding orientation. Hydroxamic acid moieties of C1 and C2 have shown interactions with functionally important metal ion and active site amino acids. The GOLD fitness scores for C1 and C2 at the active sites of two different inhibitor-induced conformations of HDAC8 were 65.658, 53.291 and 73.111, 56.362, respectively. Thus, compounds scoring GOLD fitness scores greater than 53 and 56 at C1 and C2 bound active site, respectively, were selected for further analysis on binding modes and detailed molecular interactions with the important amino acid residues. This analysis has shown that C1 has bound the active site well with its hydroxamic acid moiety contacting the catalytic machinery of HDAC8 enzyme. The carbonyl group of this hydroxamic acid which was mapped over the only HA feature of Pharm-A has formed a co-ordinate bond with metal ion. The NH and OH groups of hydroxamic acid part of C1 that mapped over the averaged HD feature have formed hydrogen bonds with H143 and D178, respectively. The interaction through the NH and OH group with H143 was not formed in C2-induced conformation of the enzyme as this H143 was a bit away from its position (Figure 10a). In terms of binding analysis of C2, a co-ordinate bond was formed between carbonyl group of the hydroxamic acid moiety which was mapped over the only HA feature of Pharm-B and metal ion whereas its hydroxyl group mapped over the average HD feature has formed a hydrogen bond with D178 (Figure 10b). The phenyl ring of C1 was placed parallel to the phenyl ring of Y306 enabling very strong π-π interaction at the surface of the tunnel-like active site. The five-membered ring of C1 was placed at the center of the tunnel to interact electrostatically with the tunnel forming hydrophobic residues. Based on the binding modes and molecular interactions observed at the active site for C1 and C2, two compounds from the database were selected as final hits of molecular docking study. Hit 1, a derivative of *N*-acetyl benzamide (Figure 11), has formed a strong hydrogen bond network with the catalytic machinery of the enzyme. The substituted amino group and fused oxygen atom of six-membered part of the fused ring of hit 1 has formed co-ordinate bond with metal ion and hydrogen bonds with D178 and H180 amino acids. The NH group of the five-membered part of the fused ring system has formed hydrogen bond with H143. The central and terminal phenyl rings were located at the middle and surface of the tunnel-like active site enabling strong hydrophobic interactions with tunnel and surface forming residues. As a part of these hydrophobic interactions, a π-π interaction was observed between the five-membered part of the fused ring of hit 1 and imidazole ring of H180 (Figure 10c). Hit 2, which is a derivative of acetamide (Figure 11), has also formed strong molecular interactions at the active site. The terminal amide moiety of hit 2 has formed a co-ordinate bond with metal ion and hydrogen bonds with H142 and D178 residues. In addition, the oxygen atom of substituted ethoxy group of a phenyl ring has formed a hydrogen bond with hydroxyl group of Y306. The three aromatic rings of this hit were placed in a way that they can strongly interact with tunnel and surface forming hydrophobic residues (Figure 10d). The fit values of hit 1 and 2 over Pharm-A and B, respectively, were 3.316, 3.245 and 2.817, 2.628 whereas the GOLD fitness scores were 72.095, 62.158 and 62.930, 53.898. Both of these compounds were identified by both the pharmacophore models and thus contain characteristics of both C1 and C2 inhibitors. Finally the novelty of these hit compounds was confirmed using *SciFinder Scholar* and *PubChem* search that these hits were not reported for HDAC8 inhibition elsewhere earlier [37,38].

## 3. Experimental Section

### 3.1. Selection of Protein and Inhibitor Structures

To date 18 crystal structures were determined by various research groups among which only 8 are native HDAC8 bound with different inhibitors [23,39–42]. Remaining structures were found to be mutant variants or bound with the same inhibitor but determined by different experimental methods or placed with different divalent metal ions at the active site (Table 2). From the 8 crystal structures available with bound inhibitors, 2V5X (PDB code) was selected based on the resolution and the topology of the active site. Despite of being determined at high resolutions, crystal structures 1T64 and 1 VKG were not used in this study because of the unusual binding characteristics of trichostatin observed in 1T64 and the gap due to the missing amino acids in 1VKG. An extensive literature search was performed over all scientific resources including patents to collect experimentally tested most potent HDAC8 inhibitors available so far. A hundred HDAC8 inhibitors tested using the same biological assay protocol were identified and two most active compounds (C1 and C2) with the IC_50_ values of 8 and 10 nM (Figure 2) were selected to be used in this study [43,44].

### 3.2. Molecular Docking and Dynamic Simulations

The selected crystal structure was checked for missing amino acids and bad valences. The water molecules and other molecular fragments present in the crystal structure were removed after checking for their catalytic importance. Prior to MD simulations, the selected most active inhibitors were docked into the active site of HDAC8 (PDB code: 2V5X) using GOLD program. The best docking pose was selected for each inhibitor based on the GOLD fitness score and molecular interactions observed with catalytic residues involved in deacetylation mechanism. The MD simulations were successfully used to verify or to generate the reliable binding mode of the docked small molecules [45]. Thus three 5 ns MD simulations were performed including an apo-form of HDAC8 and two HDAC8-inhibitor complexes using GROMACS 4.0.5 package running on a high performance linux cluster computer [46,47]. The GROMOS 96 forcefield was applied for all MD simulations [48]. The topology file of inhibitors consistent with GROMOS 96 force field was generated (supplementary file A1) by the public access PRODRG online server [49]. The molecular structures were solvated in a cubic water box of 1 nm from the surface of the structure with SPC3 water model followed by the addition of counter-ions to neutralize the system. The entire system was subjected to energy minimization using steepest descent algorithm for 10,000 steps. This energy minimization was followed by a 100 ps position restrained MD run where only the solvent molecules and counter-ions were allowed to move by fixing the protein backbone. Finally the equilibrated systems were subjected to 5 ns production runs with a time step of 2 fs at constant temperature (300 K) and pressure (1 atm). All simulations were run under periodic boundary conditions with NPT ensemble and V-rescale thermostat [50]. The structures from the dynamic trajectory were stored every 1 ps. The van der Walls (vdw) forces were treated by using a cutoff of 14 Å with the particle mesh Ewald method setting used for long-range electrostatic interactions [51]. All the post-dynamic analyses of the trajectories were done using the auxiliary analysis package available in GROMACS program.

### 3.3. Clustering of Snapshots

We used g_cluster analysis protocol available with GROMACS distribution package to find few representative snapshots from the bunch of 5000 frames saved after every 5 ns MD simulation. This clustering analysis was considered critical as the representative snapshots can represent the conformational flexibility of the protein throughout the simulation. The root mean square deviation (RMSD) after fitting or RMSD of atom-pair distance can be used to define the distance between structures. The g_cluster protocol clusters the structures by several different methods such as single linkage, Jarvis Patrick, Monte Carlo, diagonalization and gromos methods. In this study, single linkage method that adds a structure to a cluster when its distance to any element of the cluster is less than the cutoff value was used. Clusters of complete structures were generated using this method with a cutoff value of 0.077 nm.

### 3.4. Structure-Based Pharmacophore Generation and Validation

Two structural snapshots taken from two HDAC8-inhibitor complexes were considered in this step to generate structure-based pharmacophore models. Apo-form structure was not considered in pharmacophore generation as this study mainly accounts the conformational changes of protein structure due to the inhibitor binding. *Interaction generation* protocol available in Accelrys Discovery Studio 2.5 (DS) [52] was used to extract all the available hydrophilic and lipophilic interaction points that can be complemented by the inhibitor. The identified hydrogen bond acceptor (HBA), hydrogen bond donor (HBD) and hydrophobic (HY) features were clustered and edited using *Edit and Cluster Pharmacophore* tool which is available in DS. The final edited pharmacophore model comprises the necessary pharmacophoric features, which are expected to be present in the small molecule inhibitor. The final pharmacophore model was subjected to validation based on the active site orientation of inhibitors and using a data set containing 100 chemical compounds tested for HDAC8 inhibition using same biological assay protocol [53–59].

### 3.5. Database Screening

The generated structure-based pharmacophore models within DS were used as a 3D query in database searching. This virtual screening of chemical databases was conducted to find novel and diverse virtual leads suitable for further development. Database searching offers the advantage that the retrieved compounds are usually more easily available for testing than those based on *de novo* design methods [60–62]. All screening experiments were performed using the *Ligand Pharmacophore Mapping* protocol with the *Best Flexible Search* option as available in DS. A molecule must be able to map all of the features of the pharmacophore model to be listed as a hit. Geometric fit values are calculated for every database hit based on how well the chemical substructures of a compound map on to the pharmacophoric feature location constraints and their distance deviation from the feature centers. The hit compounds scoring a fit value higher than the most active compound are chosen for further studies. Hit compounds that scored fit values better than any of the most active compounds of this dataset were selected and checked for their drug-likeness properties based on Lipinski’s rule of five using DS [63]. Hit compounds those passed all of these screening tests were selected and used in molecular docking. A Lipinski-positive compound has (i) a molecular weight less than 500; (ii) less than 10 hydrogen bond acceptor groups; (iii) less than 5 hydrogen bond donor groups; and iv) an octanol/water partition co-efficient (LogP) value less than 5.

### 3.6. Molecular Docking and Lead Identification

Compounds that were predicted to be positive in Lipinski drug-likeness screening were subjected to molecular docking studies. The GOLD uses a genetic algorithm to dock the small molecules into the protein active site [64]. GOLD allows for a full range of flexibility for the ligands and partial flexibility of the protein. The performance of GOLD program upon a huge diverse set of protein-ligand complexes has been evaluated in various studies, which proved that GOLD program performs better compared to many other currently available docking programs [65,66]. Protein coordinates from the structural snapshots taken from the MD simulation trajectories were used to define the active site. The active site was defined with 10 Å radius around the bound inhibitor. The ten top-scoring conformations of every ligand were saved at the end of the calculation. Early termination option was used to skip the genetic optimization calculation when any five conformations of a particular compound were predicted within an RMSD value of 1.5 Å. The GOLD fitness score is calculated from the contributions of hydrogen bond and van der Waals interactions between the protein and ligand, intramolecular hydrogen bonds and strains of the ligand [64,67]. Protein-ligand interactions including π-π interactions were analyzed using DS and *Molegro Molecular Viewer* programs [68].

## 4. Conclusions

In order to develop dynamic structure-based pharmacophore models, we selected two of the most active HDAC8 inhibitors with considerable chemical diversity and docked into the active site of the enzyme. The best binding conformations of these compounds were selected and their complexes with HDAC8 were used in 5 ns MD simulations. After examining the results of these MD simulations, middle structures from highly clustered conformational space were obtained and used in structure-based pharmacophore model development. Two pharmacophore models, namely, Pharm-A and Pharm-B were developed considering catalytically important amino acid residues as expected inhibitor-interacting points. These pharmacophore models, as developed using structures from MD simulations that provide information over 3D conformational changes, are highly reliable compared to that of single conformation structure-based pharmacophore models. These pharmacophore models were further validated using an experimentally tested HDAC8 inhibitors dataset, which additionally strengthened the quality of the pharmacophore models. The Asinex database containing huge sets of diverse chemical compounds was virtually screened for compounds that match the pharmacophoric features identified in the study. The hit compounds were further filtered for drug-like properties based on Lipinski’s rule of five. The hits identified with drug-like properties were docked into the active site of HDAC8 enzyme to investigate the binding modes and molecular interactions formed with catalytic amino acid residues as a positive indication of enzyme inhibition. A post-docking analysis based on binding modes and molecular interactions produced two compounds as final hit compounds to be utilized in future HDAC8 inhibitor design.

## Supplementary Material



## Figures and Tables

**Table 1:** Results of validation process using HDAC8 dataset and generated pharmacophore models, Pharm-A and Pharm-B.

HDAC8 Dataset	Total	Pharm-A		Pharm-B		Pharm-X	

		Screened	Percentage (%)	Screened	Percentage (%)	Screened	Percentage (%)
Total	100	52	52	57	57	18	18
Active <0.1 μM	17	15	88.24	13	76.47	9	52.94
Moderately active ≥0.1 <1 μM	68	35	51.47	40	58.82	8	11.76
Inactive >1 μM	15	2	13.33	4	26.66	1	6.66

**Table 2:** List of crystal structures of HDAC8 determined to date.

PDB ID	Resolution (Å)	Bound Ligand	Mutation	Metal Ion
1T64	1.90	Trichostatin A	-	Zn^2+^
1T67	2.31	M344	-	Zn^2+^
1T69	2.91	SAHA	-	Zn^2+^
1VKG	2.20	CRA-19156	-	Zn^2+^
1W22	2.50	NHB	-	Zn^2+^
2V5W	2.00	Substrate	-	Zn^2+^
2V5X	2.25	V5X	-	Zn^2+^
3EW8	1.80	M344	D101L	Zn^2+^
3EWF	2.50	Substrate	H143A	Zn^2+^
3EZP	2.65	M344	D101N	Zn^2+^
3EZT	2.85	M344	D101E	Zn^2+^
3F06	2.55	M344	D101A	Zn^2+^
3F07	3.30	APHA	-	Zn^2+^
3F0R	2.54	Trichostatin A	-	Zn^2+^
3MZ3	3.20	M344	-	Co^2+^
3MZ4	1.85	M344	D101L	Mn^2+^
3MZ6	2.00	M344	D101L	Fe^2+^
3MZ7	1.90	M344	D101L	Co^2+^
