# Supplementary material for: Dynamic Structure-Based Pharmacophore Model Development: A New and Effective Addition in the Histone Deacetylase 8 (HDAC8) Inhibitor Discovery
Source: Int J Mol Sci. 2011 Dec 19;12(12):9440–62. doi: 10.3390/ijms12129440 (PMC3257139; doi:10.3390/ijms12129440)

Supplementary

Figure S1. Molecular docking of C1 and C2 at the active site of HDAC8. (**a**) The overlay of best docked poses of C1 and C2 with the full structure of HDAC8. Close views of the docked pose of C1 (**b**) and C2 (**c**). This figure was generated using Discovery Studio.

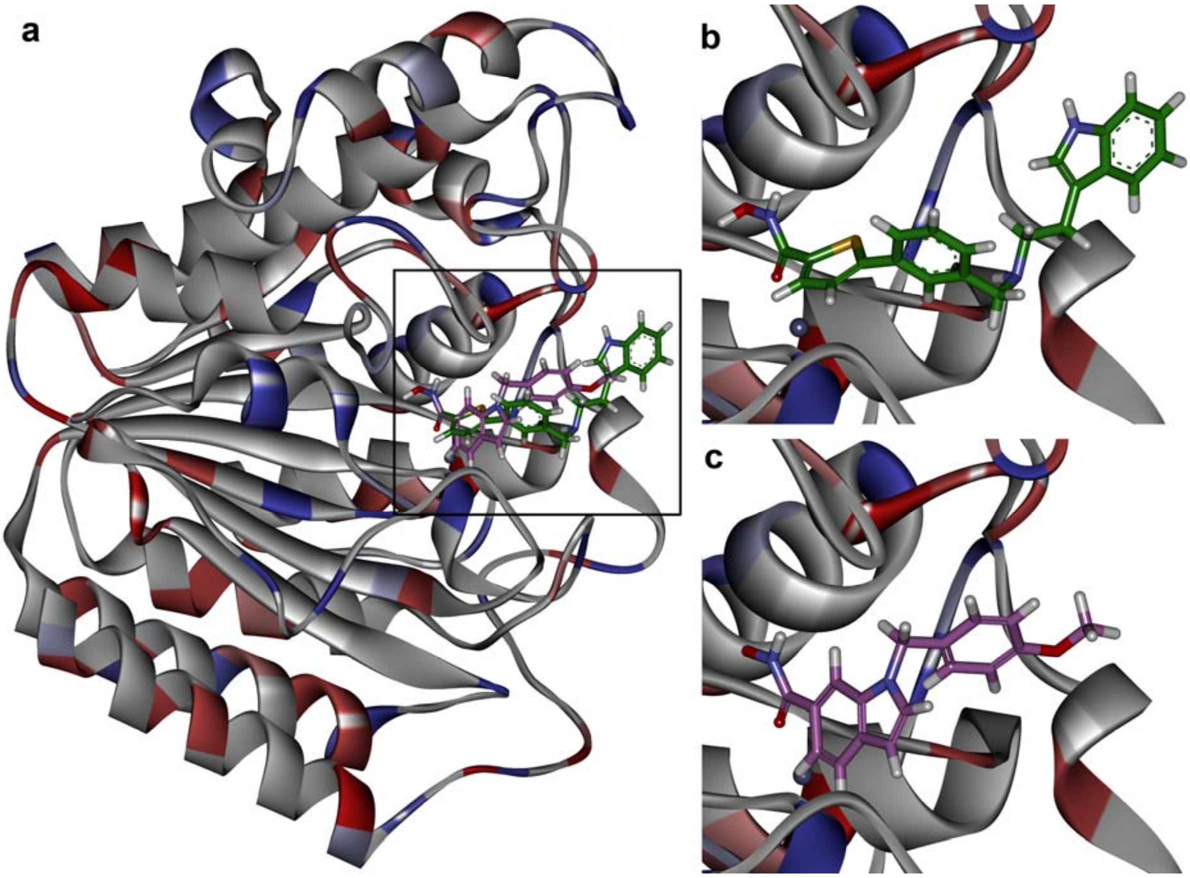

Figure S2. Distance between the hydroxamic moieties of C1, C2 and divalent metal (Zn^{2+}) ion.

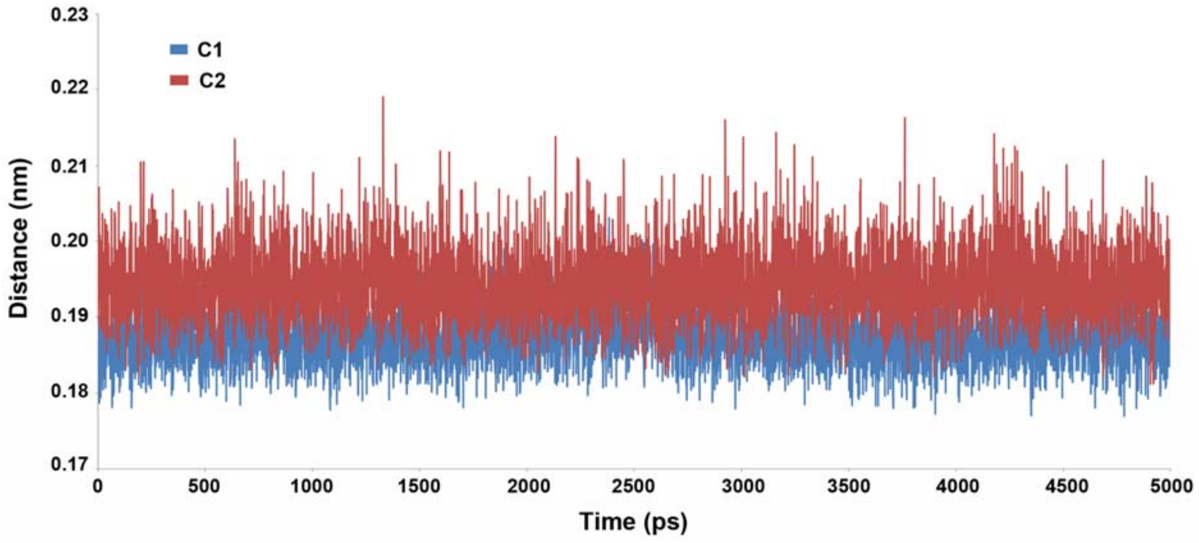

Figure S3. The number of hydrogen bonds formed between inhibitors (C1 and C2) under study and the protein.

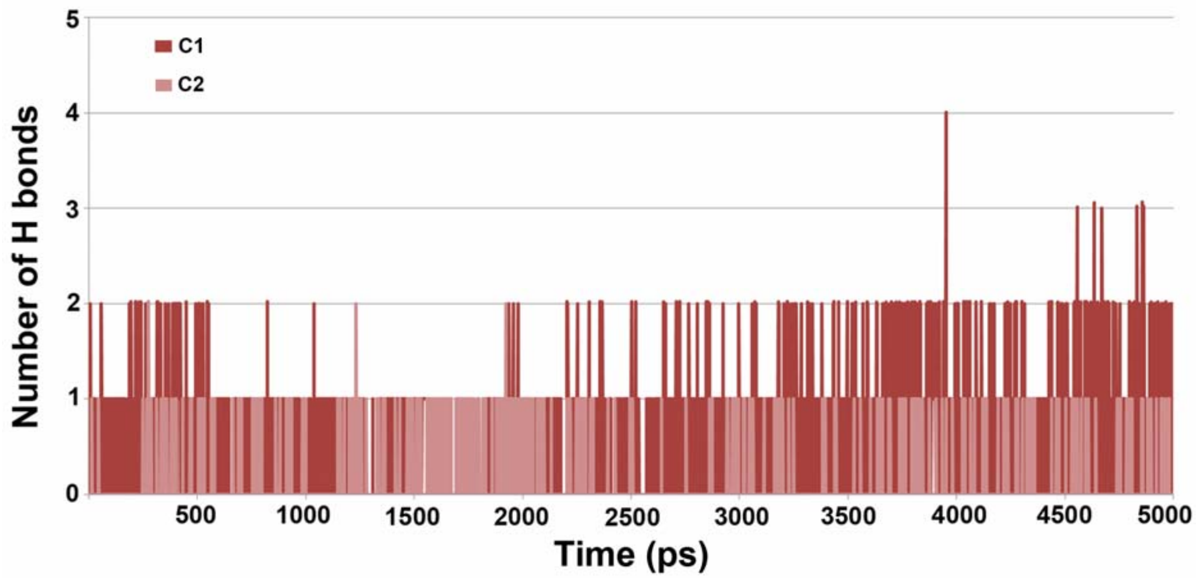

Figure S4. Pharmacophore overlay of C1 over Pharm-A shown at the active site of the dynamic HDAC8-C1 complex.

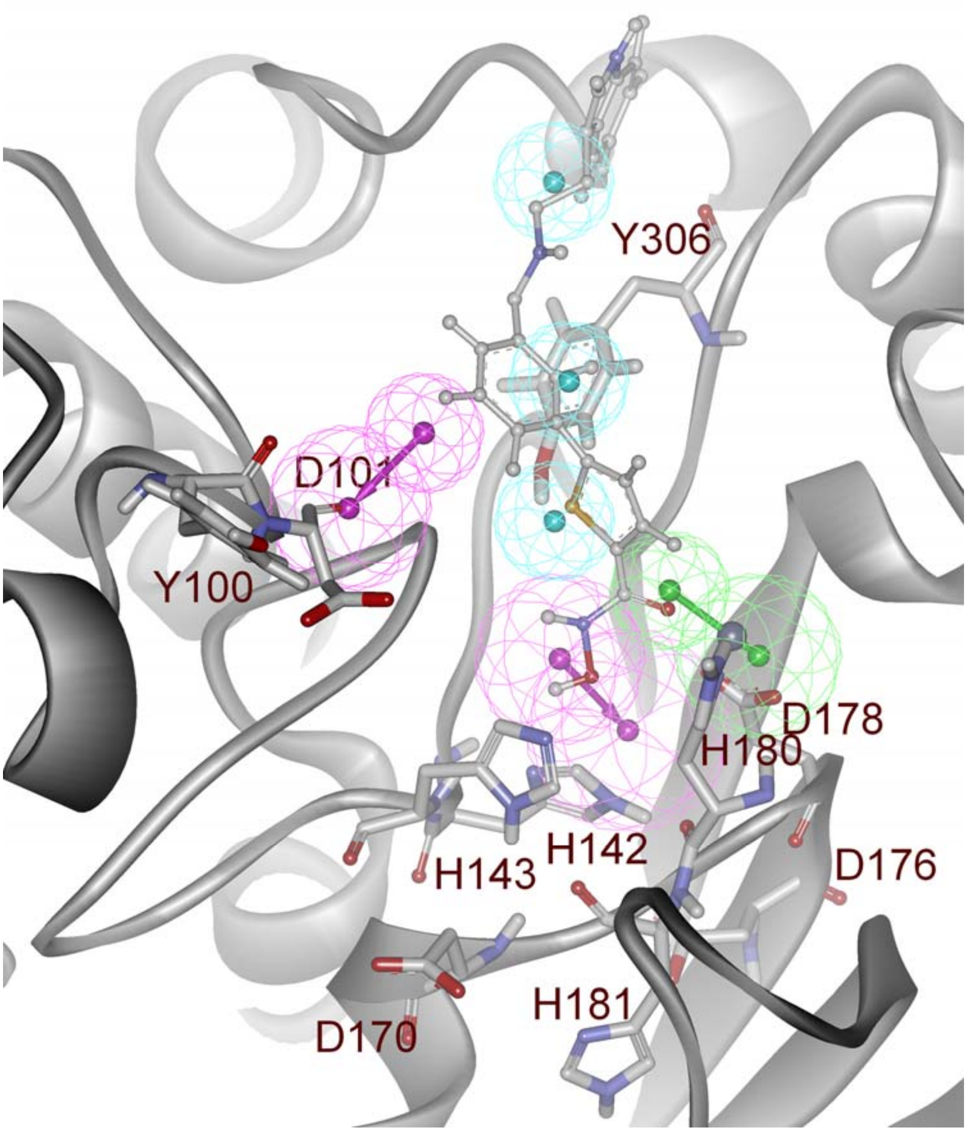

Figure S5. Pharmacophore overlay of C2 over Pharm-B shown at the active site of the dynamic HDAC8-C2 complex.

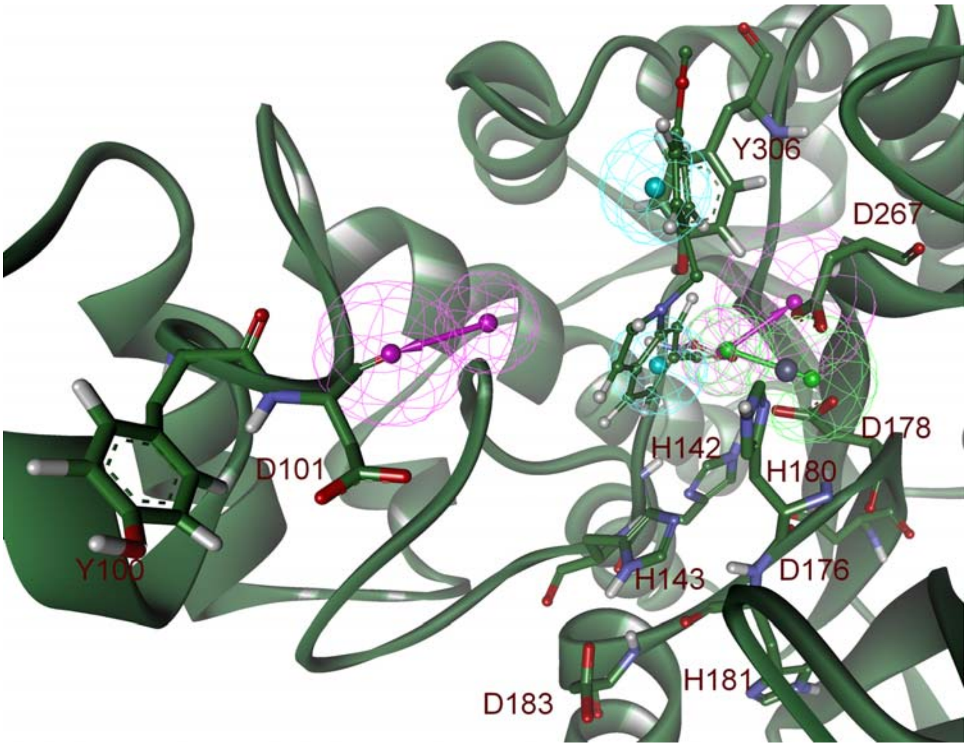

Supplement: Supplementary file 1 [file ijms-12-09440-s001.pdf]
